# Wet-chemistry processing of powdery raw material for high-tech ceramics

**DOI:** 10.1186/1556-276X-7-58

**Published:** 2012-01-05

**Authors:** Elena A Trusova, Kirill V Vokhmintcev, Igor V Zagainov

**Affiliations:** 1 Laboratory of Functional Ceramics, A.A. Baikov Institute of Metallurgy and Materials Science, Russian Academy of Sciences, Leninsky pr. 49, Moscow, 119991, Russia

## Abstract

The purpose of this study was to develop wet-chemistry approaches for the synthesis of ultradispersed and mesoporous metal oxide powders and powdery composites intended for usage in the production of ceramic materials with desired properties. The focus is on the development of template synthesis of mesoporous metal silicates as well as obtaining nano- and subnanopowders by a modified sol-gel technique and template methods. Families of mesoporous (2 to 300 nm) metal silicates and nano-oxides and subnanopowders (4 to 300 nm) were synthesized by the template method and modified sol-gel technique, respectively. Texture and morphology of the obtained objects have been studied by X-ray diffraction, scanning electron microscopy, transmission electron microscopy, Fourier transform infrared spectroscopy, Brunauer-Emmett-Teller analysis, and N_2 _adsorption-desorption. It was found that morphological parameters of the metal oxide obtained by the modified sol-gel technique depend nonlinearly on the initial molar ratio value of the sol stabilizer and metal in the reaction medium as well as the nature of the stabilizer. It has been shown that the nature of structure-directing components determines the morphology of the silicate obtained by the template method: dispersion and shape of its particles. The developed laboratory technology corresponds to the conception of soft chemistry and may be adapted to the manufacture of ultradispersed materials for catalysis, solar cells, fuel cells, semiconductors, sensors, low-sized electronic devices of new generation, etc.

## Introduction

In the last two decades, the wet-chemistry methods became the most promising commercial approaches. A specific feature of wet chemistry is the use of liquid phases: aqueous and organic solutions as well as aqueous-organic mediums [1]. Wet-chemistry approaches allow the control of the particle growth and pore structure parameters of materials up to several nanometers. To obtain materials with desired physicochemical properties, in the course of synthesis, it is necessary to carefully control the following process parameters: stirring rate, concentration of components and their quantitative ratio, electrical conductivity, density, pH, process temperature, viscosity, and other parameters which described the state of the liquid mediums. These techniques could be considered as soft chemistry with a good reason: because they do not need high temperature and pressure, they do not need to use a large number of expensive energy carrier for their technological realization and can be attributed to ecotechnology. In the technology marketplace, wet-chemistry methods have the ability to obtain nanoparticles with a narrow size distribution, to form the coatings with a controlled particle packing, and to design new-generation catalysts with high phase purity and chemical homogeneity at the atomic level. The purpose of this study was to develop wet-chemistry approaches for the synthesis of ultradispersed and mesoporous metal oxide powders and powdery composites intended for usage in the production of ceramic materials with desired properties. The focus is on the development of the template synthesis of mesoporous metal (Al, Ge, Fe, Ni, Ti, and Zr) silicates as well as obtaining the nano- and subnanopowders by a modified sol-gel technique and template methods. In these methods, it is possible to control the growth of particles in a colloid, their form, and size by changing the mentioned parameters. The dendrimer-assisted method is well known to obtain nanoparticles with a narrow size distribution [2,3]. In our research, the concept of three-dimensional [3D] oligomeric template formation *in situ *was used and developed for wet-chemistry synthesis in aqueous-organic mediums.

## Methods

Wet methods of producing nanostructured raw materials win the increasing confidence of material scientists and technologists. Their implementation is based on the use of aqueous and organic solutions and aqueous-organic fluids. Using the wet method provides control over the structure of the resulting materials at the nanoscale size. A simple adaptation of wet methods to the fabrication conditions makes them promising for a wide range of materials for spintronics, fuel cells, solar cells, implants, medical equipment, catalysts, and fine grain ceramics.

The salts of mineral acids (chlorides, nitrates, and sulfates) and organic derivatives (alcoholates or acetylacetonates) of metals were used as sources of metals. Silicic acid or tetraethoxysilane was used as a source of silicium. Hexamethylenetetramine [HMTA], *N*,*N*-dimethyloctylamine [DMOA], monoethanolamine [MEA], and tetraethylammonium hydroxide [TEAH] were used as templates or sol stabilizers [St]. The different St were used with different molar ratio values of St/metal which were varied in a wide range from 1 to 20. Syntheses were carried out in aqueous-organic mediums (deionized water, alcohols). The obtainment of mesoporous metal silicates by the template method was realized in an autoclave at 80°C to 150°C and at an autogenous pressure by stirring. Texture and morphology of the obtained powders have been studied by X-ray diffraction [XRD], scanning electron microscopy [SEM], transmission electron microscopy [TEM], Fourier transform infrared [FTIR] spectroscopy, Brunauer-Emmett-Teller [BET] analysis, and N_2 _adsorption-desorption.

## Result and discussion

We have developed (1) a template method to obtain mesoporous (2 to 30 nm) metal silicates, in which a part of silicium ions were substituted isomorphically by metal ions in the SiO_2 _lattice and (2) a modified sol-gel synthesis of nanosized (≥ 4 nm) powdery oxides of group II-VIII metals with a BET surface area ranging from a few to 150 m^2^/g. *In the case of the template method*, a catalytic co-solvolysis of organic and inorganic derivatives of silicium and metal was carried out in aqueous-organic mediums. The formation of a 3D oligomeric gel intermediate occurs from low-molecular components of the reaction mixture: co-solvents, structure-directing agents, and ligands, previously included in the composition of the metal and silicium derivatives. OH^- ^or H^+ ^groups were catalysts of the formation of oligomeric organic-inorganic gel intermediates.

*In the case of the modified sol-gel technique*, syntheses were carried out by the use of N-containing structure-directing components, which promote sol stabilization and formation of phase interface boundaries. In both cases, a thermo-treatment schedule of the obtained gel intermediate is of great importance for structure formation.

### Template method

**Figure 1:**
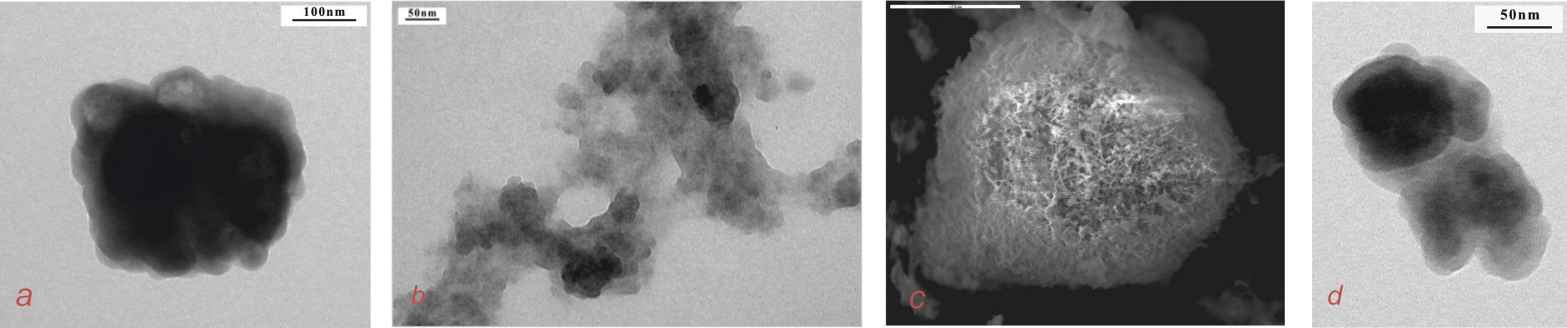
**Microphotos of Ti silicates**. (**a**) Silica obtained by base catalysis (TEM), (**b**) silica obtained by acid catalysis (TEM), (**c**) Ti_0.03_Si_0.97_O_2 _obtained by base catalysis (SEM), and (**d**) Ti_0.03_Si_0.97_O_2 _obtained by base catalysis (TEM).

**Figure 2:**
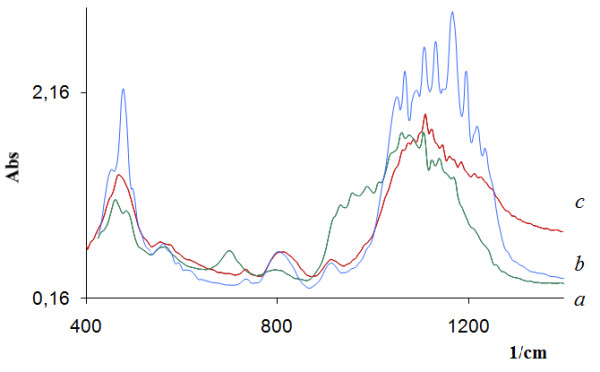
**FTIR spectra of Ti (a), Ge (b), and Fe (c) silicates calcined at 500°C**.

**Figure 3:**
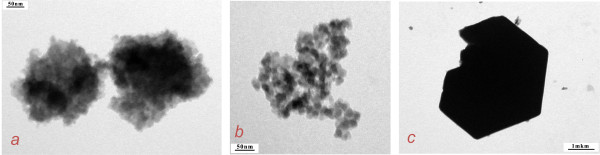
**TEM images of Al_0.25_Si_0.75_O_2+δ_**. TEM images of Al_0.25_Si_0.75_O_2+δ _synthesized using the following structure-directing agents: ammonium hydroxide (**a**), HMTA (**b**), and ammonium chloride (**c**).

The key point of the template method is the interaction between globules formed around the metal and silicium ions in the 3D template. It has been shown that in the presence of the OH^- ^or H^+ ^groups as catalysts, 3D oligomeric organic-inorganic gels can be formed by different means. As a result of these differences, the formation of silicates with a different morphology was realized. We compared the structures of silica samples obtained by base and acid catalyses. Mesoporous structures consisting of hexagonal nanocrystals with a side size of about 50 to 70 nm were obtained by base catalysis (Figure 1a). In the case of acid catalysis, calcined silica has a mesoporous structure and consists of spheres with diameters of about 20 to 40 nm (Figure 1b). The titanium silicate Ti_0.03_Si_0.97_O_2 _obtained by base hydrolysis consisted of granules with sizes of 30 to 50 μm (Figure 1c) which consist of nanotubes with outside diameters of 40 to 60 nm (Figure 1d). In obtained metal silicates, some silicium ions were isomorphically substituted by metal ions. This fact was confirmed by FTIR measurement (Figure 2), which shows a shift of characteristic bands that corresponded to the asymmetric silanol group (region 1,000 to 1,200 cm^-1^) Si-OH in the greater wave number region with an increase of the metal atom size. The comparison of microphotos in Figure 3 shows that substitution of a template by another one leads to changes in the architecture of formed globules in the metal-silicium gel and eventually to changes in the morphology of obtained aluminum silicates.

### A modified sol-gel method

In the development of a modified sol-gel technique, the choice of N-containing compounds (DMOA, HMTA, MEA, TEAH) was stipulated by several reasons: the use of small molecules in the synthesis of nanostructured objects by a 'bottom up' approach gives an ability to simulate the structure at the atomic and molecular levels; a mechanism of sol stabilization by DMOA is unknown; and there is no report on the use of DMOA for sol stabilization in the literature, but from an economic point of view, it is very attractive because of its low market value.

The choice of acetylacetone as a complexing agent was due to the fact that it forms into metal acetylacetonates which are insoluble in water. Consequently, the proposed method of obtainment can be used for the preparation of a large number of metal oxide nanopowders. Methanol and ethanol were used as co-solvents for hydrosol stabilization.

Table 1 shows the crystallite size of obtained nano- and subnanopowders calculated by the Scherrer formula. The lattice microstrain, as a rule, did not exceed 0.2% (0.04% to 0.05%) according to XRD patterns. On the example of CeO_2_, the influence of the sol St's nature on the morphology of the powders was shown. Comparison of the crystallite sizes of ceria powders shows that the use of DMOA and TEAH allows obtaining ceria powders with a little difference in crystallite sizes (12.0 and 14.5 nm, respectively). However, the BET area of ceria obtained by the use of DMOA is 1.5 times as large as the one obtained by the use of TEAH. The replacement of DMOA and TEAH by MEA resulted in an increase in the crystallite size by 1.5 to 2.0 times and as a consequence, the decrease in the surface area by three to four times.

**Table 1:** Obtained nanopowders, crystallite size (according to XRD data), and their perspective purposes

Powders	Crystallite size (nm)	Perspective purposes
Al_2_O_3_	≤ 7	Catalysts for petrochemistry (FTS, obtainment of alcohols, HDS), medicine materials (for endoprosthesis), laser equipment
Bi_2_O_3_	110 to120	Varistor ceramics, hybrid conductivity membranes
CeO_2_	7 to 60	Fuel cells, environmental catalysis (CO oxidation, HC, and soot)
Co_2_O_3_	30 to 170	Catalysts for petrochemistry (FTS, obtainment of alcohols, HDS), varistor ceramics
Cr_2_O_3_	30 to 80	Varistor ceramics
MgO	30 to 40	Medicine materials (for endoprosthesis)
MoO_x_	160 to 300	Catalysts for petrochemistry (FTS, obtainment of alcohols, HDS)
NiO	4 to 10	
WO_x_	90 to 100	
ZnO	20 to 30	Laser equipment, varistor ceramics
Ce_x_Zr_1-x_O_2_	8 to 18	Medicine materials (for endoprosthesis), hybrid conductivity membranes
CuO-CeO_2_	7 to 10	CO, HC, and soot oxidation catalysts

FTS, Fischer-Tropsch synthesis; HDS, hydrodesulfurization.

**Figure 4:**
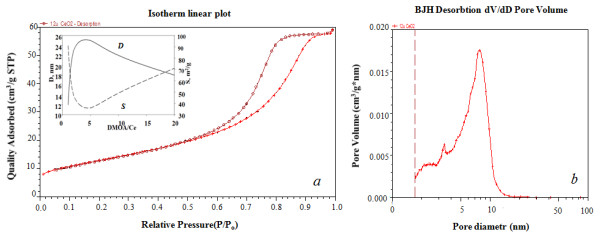
**N_2 _adsorption-desorption curves and pore size distribution**. N_2 _adsorption-desorption curves (**a**), the insert shows the impact of the amount of surfactants on the crystallite size (D) and BET area (S). Pore size distribution for the CeO_2 _powder (**b**).

Figure 4a shows the typical hysteresis loop form of N_2 _adsorption-desorption curves for CeO_2 _which corresponds to type IV and is a characteristic of mesopores, while a large part of the pore volume is provided by mesopores with a 5- to 10-nm diameter (Figure 4b). On the DMOA example, it was shown as far as the CeO_2 _morphology is highly sensitive to the initial molar ratio value of St/Ce in the reaction medium. So, the increase of this value from 1 to 5 leads to an increase in the crystallite size by two times with a simultaneous decrease in the BET area by 2.5 times. However, a further increase in this ratio results in a reverse effect (insert of Figure 4a).

## Conclusions

A complex wet-chemistry-based approach is developed for obtaining nanostructured powdery materials for different assignments. The developed method allowed obtaining the mesoporous oxides and silicates of metals with high crystallinity and given morphological parameters. Morphological parameters of the metal oxide obtained by the modified sol-gel technique depend nonlinearly on the initial molar ratio value of the sol St and metal in the reaction medium as well as on the nature of the St. The nature of structure-directing components determines the morphology of the silicate obtained by the template method: dispersion and shape of its particles. The developed laboratory technology corresponds to the conception of soft chemistry and may be adapted to manufacture ultradispersed materials for catalysis, solar cells, fuel cells, semiconductors, sensors, low-sized electronic devices of new generation, etc.

## Competing interests

The authors declare that they have no competing interests.

## Authors' contributions

EAT conceived the study, was responsible for its coordination and the interpretation of results, and drafted the manuscript. KVV and IVZ carried out the synthesis of metal silicates and metal oxides as well as participated in the interpretation of the experimental results. All authors read and approved the final manuscript.
